# Integration of Genomic and Cytogenetic Data on Tandem DNAs for Analyzing the Genome Diversity Within the Genus *Hedysarum* L. (Fabaceae)

**DOI:** 10.3389/fpls.2022.865958

**Published:** 2022-04-29

**Authors:** Olga Yu. Yurkevich, Tatiana E. Samatadze, Inessa Yu. Selyutina, Natalia A. Suprun, Svetlana N. Suslina, Svyatoslav A. Zoshchuk, Alexandra V. Amosova, Olga V. Muravenko

**Affiliations:** ^1^Engelhardt Institute of Molecular Biology, Russian Academy of Sciences, Moscow, Russia; ^2^Peoples’ Friendship University of Russia, Moscow, Russia; ^3^Central Siberian Botanical Garden, Siberian Branch of the Russian Academy of Sciences, Novosibirsk, Russia; ^4^Central Volgograd Regional Botanical Garden, Volgograd, Russia

**Keywords:** next-generation sequencing (NGS), *Hedysarum* L., repeatome, tandem DNAs, 5S rDNA, FISH analysis, chromosome variability, 35S rDNA

## Abstract

The section *Multicaulia* is the largest clade in the genus *Hedysarum* L. (Fabaceae). Representatives of the sect. *Multicaulia* are valuable plants used for medicinal and fodder purposes. The taxonomy and phylogeny of the sect. *Multicaulia* are still ambiguous. To clarify the species relationships within sect. *Multicaulia*, we, for the first time, explored repeatomes of *H. grandiflorum* Pall., *H. zundukii* Peschkova, and *H. dahuricum* Turcz. using next-generation sequencing technologies and a subsequent bioinformatic analysis by RepeatExplorer/TAREAN pipelines. The comparative repeatome analysis showed that mobile elements made up 20–24% (Class I) and about 2–2.5% (Class II) of their repetitive DNAs. The amount of ribosomal DNA varied from 1 to 2.6%, and the content of satellite DNA ranged from 2.7 to 5.1%. For each species, five high confident putative tandem DNA repeats and 5–10 low confident putative DNA repeats were identified. According to BLAST, these repeats demonstrated high sequence similarity within the studied species. FISH-based mapping of 35S rDNA, 5S rDNA, and satDNAs made it possible to detect new effective molecular chromosome markers for *Hedysarum* species and construct the species karyograms. Comparison of the patterns of satDNA localization on chromosomes of the studied species allowed us to assess genome diversity within the sect. *Multicaulia*. In all studied species, we revealed intra- and interspecific variabilities in patterns of the chromosomal distribution of molecular chromosome markers. In *H. gmelinii* Ledeb. and *H. setigerum* Turcz. ex Fisch. et Meyer, similar subgenomes were detected, which confirmed the polyploid status of their genomes. Our findings demonstrated a close genomic relationship among six studied species indicating their common origin and confirmed the taxonomic status of *H. setigerum* as a subspecies of *H. gmelinii* as well as the validity of combining the sect. *Multicaulia* and *Subacaulia* into one sect. *Multicaulia*.

## Introduction

The section *Multicaulia* is the largest clade in the genus *Hedysarum* L. (Fabaceae). The species included in this section are distributed mainly in Central Asia (Malyshev et al., 2012) and are actively used in medicine because they contain various biologically active compounds (Vysochina et al., 2011; Dong et al., 2013; Liu Y. et al., 2019). The high content of mangiferin (a natural xanthone) in *Hedysarum gmelinii* Ledeb., *H. grandiflorum* Pall., and *H. setigerum* Turcz. ex Fisch. et Meyer makes them promising raw materials for obtaining antiviral and antibacterial agents (Neretina et al., 2002; Kuprina and Lutsky, 2014; Imachuyeva et al., 2020). In addition, some *Hedysarum* species are able to provide high increases in biomass in summer, and they are valuable forage plants (Fedtschenko, 1948; Sa et al., 2010; Malyshev et al., 2012). To preserve the gene pool of rare and valuable species of *Hedysarum*, their reintroduction was performed, and also synthetic populations and *in vitro* collections were developed (Maslova et al., 2019; Avramova and Cherepanova, 2020; Suprun et al., 2020).

The taxonomy and phylogeny of the sect. *Multicaulia*, similar to the entire genus *Hedysarum*, are still ambiguous. According to phylogenetic analyses using nuclear (ITS) and plastid DNA sequences (trnL—trnF and matK), two main lineages in the genus *Hedysarum*, the *Hedysarum* s.s. clade (*Gamotion*) and the *Sartoria* clade (*Multicaulia*), are presented (Choi and Ohashi, 2003; Duan et al., 2015; Liu et al., 2017; Nafisi et al., 2019). The *Hedysarum* sect. *Multicaulia* includes three subsections *Multicaulia*, *Subacaulia*, and *Crinifera* (Choi and Ohashi, 2003). However, some taxonomists are assigned *Subacaulia* and *Multicaulia* to different sections (Fedtschenko, 1948; Malyshev et al., 2012). Investigation of species genome diversity within the genus *Hedysarum* by means of molecular AFLP and ISSR markers detected a high level of intraspecific genetic polymorphism (Marghali et al., 2005; Bushman et al., 2007; Zvyagina and Dorogina, 2013; Qiang et al., 2018). Considerable variability of morphological features observed in the species of the section *Multicaula* often prevents their accurate identification, especially in the areas where their ranges overlap. The population structure analysis in East European (*H. grandiflorum* Pall., *H. biebersteinii* Zertova, and *H. argyrophyllum* Ledeb.) and South Siberian (*H. setigerum* and *H. gmelinii*) species of the sect. *Multicaulia*, performed based on the molecular genetic (ISSR) markers, did not clearly distinguish species with overlapping ranges (Schanzer and Suprun, 2012; Zvyagina et al., 2016).

In species of the sect. *Multicaulia*, a basic chromosome number of x = 8 was mostly revealed using monochrome chromosome staining (Choi and Ohashi, 2003; Arslan et al., 2012; Zvyagina et al., 2016; Kumar et al., 2018). Besides, in the karyotype of diploid Siberian species *H. sangilense* Krasnob., supernumerary small chromosomes were detected (Krogulevich and Rostovtseva, 1984). However, for some species, such as the endemic *H. dahuricum* Turcz. ex B. Fedtsch., growing in Eastern Siberia and Mongolia (Kurbatsky, 1994), chromosome numbers have not been determined yet. The analysis of chromosome C-banding patterns, performed in the karyotype of *H. coronarium* L. originating from North-East Algeria, revealed three types of bands (terminal, intercalary, and pericentromeric) (Issolah et al., 2006). In four Algerian species, *Hedysarum carnosum* Desf., *H. spinosissimum* L., *H. pallidum* Desf., and *H. naudinianum* Coss., 35S rDNA loci, localized in one chromosome pair, and different numbers and position of 5S rDNA clusters (in one or two pairs) were detected by fluorescent *in situ* hybridization (FISH) (Benhizia et al., 2021).

The taxonomic status of *H. gmelinii* and *H. setigerum*, having overlapping ranges and high morphological similarity, is still unclear, and *H. setigerum* is sometimes considered as a subspecies of closely related *H. gmelinii* (*H. gmelinii* spp. *setigerum*) or as a separate species (Fedtschenko, 1948; Kurbatsky, 1994; Sa et al., 2010). In *H. setigerum* and *H. gmelinii*, chromosome numbers varied greatly in different reports, and chromosome numbers 2*n* = 14, 16, 28, 32, 48, and 56 were revealed (Gatsuk, 1967; Plennik and Rostovtseva, 1977; Malahova and Kurbatsky, 1992; Philippov et al., 2008; Cherkasova, 2009). Recently, FISH with 35S rDNA and 5S rDNA, performed in several species of the sect. *Hedysarum*, has demonstrated that 35S rDNA and 5S rDNA clusters could be effective molecular chromosomal markers facilitating precise identification of morphologically similar species of the sect. *Hedysarum* (Yurkevich et al., 2021). Also, various tandem repeats are applied as chromosomal markers to detect intra- and interspecific diversities in plant genomes, chromosomal rearrangements, and the evolutionary pathways in various taxa, including Fabaceae species (Pamponét et al., 2019; Ávila Robledillo et al., 2020; Campomayor et al., 2021; Waminal et al., 2021). The use of such molecular chromosomal markers for karyotype analyses in species from the sect. *Multicaulia* will make it possible to specify their taxonomy, chromosome numbers, and ploidy status.

Due to the diversity of repetitive DNA sequences, plant genomes vary greatly in composition and size (McCann et al., 2020). Comparative repeatome analyses in related species provide new insight into the organization and divergence of their genomes (Liu Q. et al., 2019; McCann et al., 2020; Zwyrtková et al., 2020). For the understanding of species relationships within the sect. *Multicaulia*, further studies of genomic diversity are needed. In particular, intra- and interspecific variabilities in composition and genomic organization of transposable elements and satDNA should be explored in different *Hedysarum* species as well as in accessions from various growing areas.

To explore intra- and interspecific genome diversities and clarify the species relationships within the section *Multicaulia*, we performed a comparative characterization of repeatomes of *H. grandiflorum*, *H. zundukii* Peschkova, and *H. dahuricum*, which included the genome-wide bioinformatic analysis by RepeatExplorer/TAREAN pipelines. We also carried out FISH mapping of the identified tandem DNAs on chromosomes of these species and also three other related species of the sect. *Multicaulia* from different regions of Eurasia. Additionally, we explored available data on these species distributions within Eurasia to construct the integrated schematic map of their habitats.

## Materials and Methods

### Plant Materials

We examined thirteen plant accessions covering six *Hedysarum* species of the sect. *Multicaulia* (Choi and Ohashi, 2003) from subsections *Multicaulia* (*H. dahuricum*, *H. razoumovianum* Fisch. et Helm ex DC., *H. setigerum*, and *H. gmelinii*) and *Subacaulia* (*H. grandiflorum* and *H. zundukii*). These specimens were obtained from different seed sources (detailed in Table 1).

**TABLE 1:** The list of the studied *Hedysarum* accessions.

Species	Voucher/origin
*H. dahuricum* Turcz. ex B. Fedtsch.	ZAN27072007/50.83636° N; 114.83706°E; Nozhiy lake, Zabaikalye region, Russia/collected by Dr. I.Yu. Selyutina and Dr. N.A. Karnaukhova, 2007
*H. dahuricum* Turcz. ex B. Fedtsch.	ZAK29072007/50.83636° N; 114.83706°E; Kunkur village, Zabaikalye region, Russia/collected by Dr. I.Yu. Selyutina and Dr. N.A. Karnaukhova, 2007
*H. gmelinii* Ledeb.	AOCH22091999/50.40343° N; 86.69024°E; Altai Republic, Chuuya river, Russia/collected by Dr. N.A. Karnaukhova, 1999
*H. gmelinii* Ledeb.	ACHK13082016/51.03956° N; 86.22734° E; Altai Republic, Kuyus village, Russia/collected by Dr. I.Yu. Selyutina, 2016
*H. gmelinii* Ledeb.	NIK01082020/54.47672° N; 83.27336° E; Novosibirsk region, Koinicha river, Russia/collected by Dr. I.Yu. Selyutina, 2020
*H. gmelinii* Ledeb.	54-19/germplasm collection of the Botanical Garden of the Ammosov North-Eastern Federal University, Republic of Sakha (Yakutia), Russia, 2019
*H. setigerum* Turcz. ex Fisch. et Meyer	IOO23072005/53.41179° N; 107.78922° E; Irkutsk region, Olkhon island, Russia/collected by Dr. I.Yu. Selyutina, 2005
*H. setigerum* Turcz. ex Fisch. et Meyer	AKZ18072016/49.51919° N; 88.04097° E; Altai Republic, Zhumaly river, Russia/collected by Dr. I.Yu. Selyutina, 2016
*H. grandiflorum* Pall.	32-10/49°29′ N; 43°30′ E; Volgograd region, Russia/collected by Dr. N.A. Suprun, 2010
*H. grandiflorum* Pall.	98-11/49°34′ N; 42°11′ E; Volgograd region, Russia/collected by Dr. N.A. Suprun, 2011
*H. razoumovianum* Fisch. et Helm ex DC.	SBR20072006/52.0745° N; 51.19229°E; Samara region, Rostashi village, Russia/collected by Dr. V.N. Ilyina, 2006
*H. zundukii* Peschkova	IOZ28072005/53.4014° N; 107.41213° E; Irkutsk region, Cape Zunduk, Russia/collected by Dr. I.Yu. Selyutina and Dr. N.A. Karnaukhova, 2005
*H. zundukii* Peschkova	IOO13072007/53.3413° N; 107.2698° E; Irkutsk region, Cape Otto-Khushun, Russia/collected by Dr. I.Yu. Selyutina and Dr. N.A. Karnaukhova, 2007

Wild *Hedysarum* accessions were collected and identified by Dr. I.Yu. Selyutina and Dr. N.A. Karnaukhova [the Central Siberian Botanical Garden (CSBG), SB RAS, Russia], and also Dr. N.A. Suprun [Volgograd Regional Botanical Garden (VRBG), Volgograd, Russia] (Table 1).

### Construction of Schematic Map of Species Distribution Areas

For the studied species from the sect. *Multicaulia*, an integrated schematic map of their distribution within the northern, central, and eastern parts of Eurasia was constructed based on the analysis of currently available data (Fedtschenko, 1948; Malyshev and Peshkova, 1984; Kurbatsky, 1994; Bardunov et al., 2006; Wu et al., 2010; Malyshev et al., 2012).

### Sequence Analysis and Identification DNA Repeats

Genomic DNAs of *H. grandiflorum*, *H. zundukii*, and *H. dahuricum* were isolated from young leaves using the CTAB method with minor modifications (Rogers and Bendich, 1985). Genome DNA low-coverage sequencing was performed at the Beijing Genomics Institute (BGISeq platform) (Shenzhen, Guangdong, China) according to the NGS protocol for generating 5–10 million of paired-end reads of 150 bp in length, which was at least 0.5–0.9x of the coverage of the *Hedysarum* genome (1C = 1,643 Mbp, Benhizia et al., 2021). The raw data were uploaded to the NCBI database.^1^ The comparative integrated bioinformatic analysis of repeatomes of *H. grandiflorum, H. zundukii*, and *H. dahuricum* was performed using RepeatExplorer/TAREAN pipelines (Novak et al., 2013, 2017). For each studied species, the genomic reads were filtered by quality, and then 1,000,000 high-quality reads were randomly selected for further analyses, which corresponds to 0.09x of coverage of the genome *Hedysarum* (1C = 1,643 Mbp, Benhizia et al., 2021) and is within the limits recommended by the developers of these programs (genome coverage of 0.01–0.50x is recommended) (Novak et al., 2017). RepeatExplorer/TAREAN was launched with the preset settings based on the Galaxy platform.^2^ The default threshold is explicitly set to 90% sequence similarity spanning at least 55% of the read length (in the case of reads differing in length it applies to the longer one). The sequence homology of the identified tandem DNA repeats was estimated by Basic Local Alignment Search Tool (BLAST) (NCBI, MD, United States). A number of seven abundant tandem DNA repeats of *H. zundukii*, which exhibited high sequence homology with five DNA repeats of *H. grandiflorum* and six DNA repeats of *H. dahuricum*, were used for generating oligonucleotide FISH probes (refer to Supplementary Table 1) by Primer3-Plus software (Untergasser et al., 2007).

### Chromosome Spread Preparation

Seeds of the studied *Hedysarum* species were sacrificed, kept in hot (60–75°C) water for 5–10, min and then germinated in Petri dishes for a week at 22°C. Excised root tips (0.5–1 cm long) were incubated in ice water for 16–24 h for accumulation of mitotic cells and fixed in ethanol/acetic acid (3:1) for 3–24 h at room temperature. Then, the roots were put into a 1% acetocarmine solution for good chromosome spreading (in 45% acetic acid) for 15 min. A root tip was cut on the slide, macerated with a dissecting needle in a drop of 45% acetic acid, and covered with a coverslip. After squashing and freezing in liquid nitrogen, the slides were dehydrated in 96% ethanol and air-dried.

### Fluorescent *in situ* Hybridization Procedure

In FISH assays, we used two wheat DNA probes pTa71 containing 18S-5.8S-26S (35S) ribosomal DNA (rDNA) (Gerlach and Bedbrook, 1979) and pTa794 containing 5S rDNA (Gerlach and Dyer, 1980). These DNA probes were labeled directly with fluorochromes Aqua 431 dUTP and Red 580 dUTP (ENZO Life Sciences, NY, United States) using Nick Translation DNA Labeling System 2.0 (Life Sciences Inc., NY, United States). Additionally, we used oligonucleotide probes Hz 2, Hz 6, Hz 9, Hz 44, Hz 59, Hz 75, and Hz 96, which were synthesized with labeled nucleotides Cy3-dUTP or 6-FAM-dUTP in *Evrogen JSC* (Moscow, Russia). Before the FISH procedure, chromosome slides were pretreated with 1 mg/ml of RNase A (Roche Diagnostics, Mannheim, Germany) in 2 × SSC at 37°C for 1 h. Then, the slides were washed three times for 10 min in 2 × SSC, dehydrated in the graded ethanol series (70, 85, and 96%) and air-dried. The hybridization mixture (22 μl) containing 40 ng of each labeled probe was added to each slide. Coverslips were placed on the slides and sealed with rubber cement. Slides with DNA probes were co-denatured at 74°C for 5 min, placed in a moisture chamber, and hybridized overnight at 37 C. After FISH, the slides were placed in 0.1 × SSC at 50°C for 5 min, 2 × SSC at 37°C for 10 min, 2 × SSC at RT for 5 min, and 1 × PBS at RT for 3 min. Then, the slides were dehydrated in the graded ethanol series, air-dried, and stained with 0.1 μg/ml DAPI (4′,6-diamidino-2-phenylindole) (Serva, Heidelberg, Germany) in Vectashield mounting medium (Vector Laboratories, Peterborough, United Kingdom).

### Chromosome Analysis

At least five plants of each accession and fifteen metaphase plates from each specimen were examined. The chromosome slides were analyzed with the Olympus BX—61 epifluorescence microscope with a standard narrow bandpass filter set (Olympus, Tokyo, Japan). Images were captured with a monochrome charge-coupled device camera (Cool Snap, Roper Scientific, Inc., Sarasota, FL, United States). Then, they were processed using Adobe Photoshop 10.0 software (Adobe, Birmingham, United States).

## Results

### Distribution Areas of the Studied *Hedysarum* Species

**FIGURE 1:**
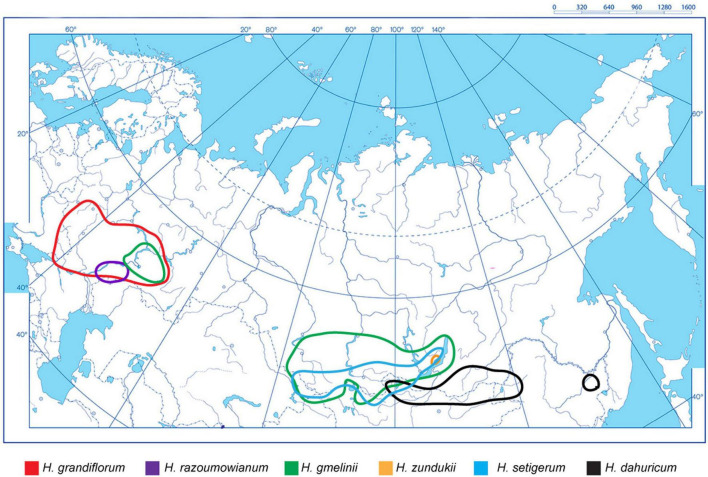
An integrated schematic map showing distribution of *H. grandiflorum*, *H. razoumovianum*, *H. dahuricum*, *H. zundukii*, *H. gmelinii*, and *H. setigerum* within the northern, central, and eastern parts of Eurasia. The species names and correspondent colors of the lines indicating the boundaries of the species occurrence are specified under the maps.

**FIGURE 2:**
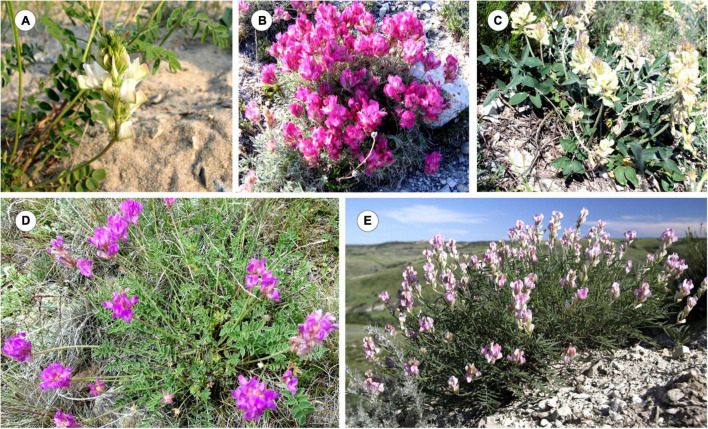
Wild populations of *H. dahuricum* (Transbaikal territory, Russia) **(A)**, *H. zundukii* (Irkutsk region, Russia) **(B)**, *H. grandiflorum* (Volgograd region, RF) **(C)**, *H. setigerum* (Irkutsk region, Russia) **(D)**, and *H. razoumovianum* (Volgograd region, Russia) **(E)**. The images were taken by I. Yu. Selyutina **(A,D)**, S. G. Kazanovsky **(B)**, N. A. Suprun **(C)**, and A. V. Popov **(E)**.

As shown on the constructed integrated schematic map of the distribution of the studied species within Eurasia (Figure 1), *H. razoumovianum* is a rare steppe subendemic species distributed in the Southern Urals and the Middle Volga Region (Fedtschenko, 1948). Another rare species *H. grandiflorum* grows along the middle and lower reaches of the Don River, in the Volga Region including the Volga Upland, in the South of the Urals, and in Kalmykia (Fedtschenko, 1948). The endemic species *H. zundukii* grows within a narrow region occupying the western coast of Lake Baikal. *Hedysarum zundukii* is considered to be an ancient relict species of desert-steppe flora (Malyshev and Peshkova, 1984). The entire range of this species (no more than 18 km long and up to 2 km wide) is limited to a section of the western coast of Lake Baikal from Cape Oto-Khushun to Cape Zama (Cape Zunduk is its classic locality), which is located in the Olkhonsky district of the Irkutsk region (Bardunov et al., 2006). *Hedysarum gmelinii* is a mountain-steppe species distributed in the southeast of the European part of Russia, and also in Siberia, partially covering the north of Kazakhstan and Mongolia (Fedtschenko, 1948; Kurbatsky, 1994). Moreover, *H. gmelinii* demonstrates considerable variability in its morphological characters; therefore, this species could potentially occupy various ecological niches (steppes, meadows, forests, river valleys, rocks, and limestones) (Peshkova, 2001). *Hedysarum gmelinii* has been found on the territory of Central and Northwestern China, where it occupies stony steppes, at an altitude of 800–1,800 m above sea level (Wu et al., 2010). *Hedysarum setigerum* is distributed in East Siberia and South Siberia, where it occupies almost the same areas as its closely related species *H. gmelinii*. Specifically, *H. setigerum* is found in the Altai Mountains, Krasnoyarsk Region, the Republic of Khakassia, Tuva, Irkutsk Region, Buryatia, and the Republic of Sakha (Yakutia) (Fedtschenko, 1948; Malyshev et al., 2012). *Hedysarum dahuricum* grows in steppes, on rocky slopes and rocks in Eastern Siberia and northern Mongolia (Kurbatsky, 1994). In Figure 2, several studied species of the *Hedysarum* sect. *Multicaulia* growing in natural habitat are presented.

### Satellite Repeat Identification by RepeatExplorer/TAREAN Pipelines

**FIGURE 3:**
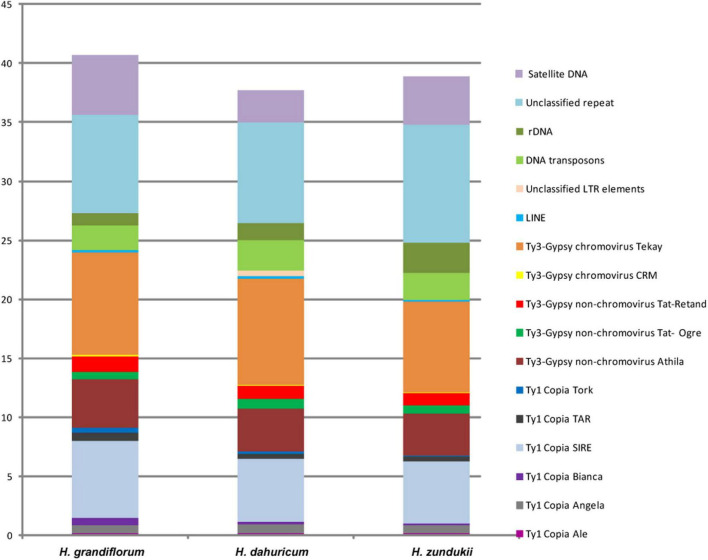
Genome proportion of most abundant DNA repeats in *H. grandiflorum*, *H. dahuricum*, and *H. zundukii*. The genome proportion of individual repeat types was obtained as a ratio of reads specific to individual repeat types to all reads used for clustering analyses by the RepeatExplorer pipelines.

DNA sequencing of the genomes of *H. grandiflorum*, *H. zundukii*, and *H. dahuricum* was carried out using NGS-based technology. According to the results of RepeatExplorer/TAREAN analysis, transposable elements (TEs) made up the majority of their repetitive DNAs (Figure 3 and Supplementary Table 2). Depending on the species, 20–24% of the revealed TEs belonged to retrotransposons (Class I), and about 2–2.5% of the TEs belonged to transposons (Class II) (with EnSpm_CACTA predominating). The long terminal repeats (LTR) retrotransposons were the most abundant mobile elements of Class I. They included 13–14.87% of Ty3-Gypsy superfamily (mostly non-chromovirus Athila, chromovirus Tekay) and 6.78–9.1% of Ty1-Copia superfamily (mostly SIRE). In *H. grandiflorum* and *H. dahuricum*, more TEs of Class I were found when compared to *H. zundukii* (Supplementary Table 2). The content of ribosomal DNA represented 1–2.6% of the genomes of the studied species. Satellite DNA makes up a small proportion of their genome (2.68–5.09%), and the largest amount was found in *H. grandiflorum*.

Using TAREAN, five high confident putative satellites per species and 5–10 (depending on the species) low confident putative satellites were determined. The largest number of putative satellites (15, in total) was found in the relict species *H. zundukii.* Among them, seven promising putative DNA satellites that could be the potential cytogenetic markers for *Hedysarum* were identified. The genome proportion of each tandem DNA repeat and other details, including their consensus length, are shown in Table 2. In *H. zundukii*, most abundant was a tandem repeat Hz 2 (homologous repeats Hd 3 and Hg10); in *H. dahuricum*, most abundant was a tandem repeat Hd 2 (homologous repeat Hz 6); and in *H. grandiflorum*, most abundant was a tandem repeat Hg 2 (homologous repeats Hz 9 and Hd 15). A total of four DNA repeats of *H. zundukii* (Hz 2, Hz 9, Hz 44, and Hz 96) and their homologous repeats in *H. dahuricum* (Hd 3, Hd 15, Hd 79, and Hd 105) and *H. grandiflorum* (Hg 10, Hg 2, Hg 74, and Hg 98) exhibited 100% of sequence identity. In *H. zundukii* and *H. dahuricum*, two DNA repeats Hz 59 and Hd 155 had almost 100% of identity but the corresponding homologous repeat was not detected in *H. grandiflorum*. Also, the homologous repeats Hz 75 and Hg 102 had 100% of sequence identity in *H. zundukii* and *H. grandiflorum* though the corresponding homologous repeat was not detected in *H. dahuricum* (Table 2).

**TABLE 2:** Homology* of tandem repeats identified in genomes of *H. grandiflorum*, *H. dahuricum*, and *H. zundukii* and FISH-based patterns of their chromosomal distributions.

Tandem repeat/genome proportion, %			Repeat length, bp	Blast homology	Chromosome localization
*H. zundukii*	*H. dahuricum*	*H. grandiflorum*			
Hz 6/1.5	Hd 2/1.6	no	2452—Hz 2982—Hd (94% identity with Hz 6)	73% identity with Cicer arietinum chromosomes CP040771.1, CP040768.1, CP039337.1, 70% identity *Lupinus angustifolius* cultivar Tanjil chromosome LG CP023118.1, CP023114.1	Subtelomeric regions of some chromosomes
Hz 9/1.2	Hd 15/0.7	Hg 2/1.9 Hg 5/1.4 (89% identity with Hg 2)	180	no	Pericentromeric regions
Hz 2/2.3	Hd 3/1.2	Hg 10/1.1	50	no	Pericentromeric regions
Hz 44/0.27	Hd 79/0.14	Hg 74/0.15	324	90% identity with *Digitaria exilis* genome assembly, chromosome: 9B, LR761622.1	Pericentromeric regions
Hz 96/0.041	Hd 105/0.064	Hg 98/0.08	1361	80% identity with *Vigna unguiculata* cultivar Xiabao 2 chromosomes CP039345.1, CP039355.1, CP039346.1	Pericentromeric regions
Hz 75/0.076	no	Hg 102/0.074	1713	70% identity with *Jasminum sambac* cultivar Hutoumoli linkage group Lg 1, 3, 10, CP073646.1, CP073640.1, CP073641.1	Weak signals in the subtelomeric regions of some chromosomes
Hz 59/0.019	Hd 155/0.023	no	1045—Hz 959—Hd (98% identity with Hz 59)	68% identity with *Medicago truncatula* chromosome 5 clone mte1-8e5, CT573053.1	Weak signals in the subtelomeric regions of some chromosomes

*By default, the repeat identity is 100%. The repeats have the same length unless otherwise stated. The names of the repeats used as FISH probes are specified in bold type.

In *H. dahuricum*, tandem DNA repeat Hd 2 (94% identity with repeat Hz 6) had the highest percent of genome proportion (2.3%). In *H. zundukii*, the tandem DNA repeat Hz 6 had the second largestgenome proportion (1.5%) among the identified repeats (Table 2). However, the homologous repeat was not detected in the repeatome of *H. grandiflorum.* The tandem DNA repeats Hz 9 (homologous to Hd 15 and Hg 2) and Hz 2 (homologous to Hd 3 and Hg 10) were the second and third largest repeats in the genomes of the studied species. The genome proportions of repeats Hz 44, Hz 96, Hz 75, and Hz 59, as well as the corresponding homologous repeats in *H. dahuricum* and *H. grandiflorum*, were considerably less. The repeats Hz 6 and Hd 105 and several other examined DNA repeats exhibited partial homology with DNA repeats identified in genomes of other Fabaceae species (detailed in Table 2). According to BLAST, the homology of repeats Hz 9 and Hz 2 with the sequences available in NCBI was not revealed.

### Karyotype Structure and Chromosomal Localization of 35S rDNA, 5S rDNA, and Satellite DNAs in the Studied *Hedysarum* Species

To determine or confirm the previously reported chromosome numbers within *Hedysarum*, we analyzed the karyotypes of all studied species. The results are presented in Table 3 along with the currently available data. In *H. dahuricum*, we determined, for the first time, the number of chromosomes (2*n* = 2x = 16). In *H. gmelinii* and *H. setigerum*, we confirmed the chromosome numbers (2*n* = 4x = 32). In one *H. gmelinii* accession, two karyotypes, diploid (2*n* = 2x = 16) and hexaploid (2*n* = 6x = 48), were revealed (Table 3).

**TABLE 3:** Chromosome numbers determined in the studied *Hedysarum* species.

Species	Chromosome number, our data	Literature data
*H. dahuricum* ZAN27072007	2*n* = 16	No data
*H. dahuricum* ZAK29072007	2*n* = 16	
*H. gmelinii* AOCH22091999	2*n* = 32	2*n* = 14; 28; 32; 48 (Malahova and Kurbatsky, 1992; Philippov et al., 2008; Cherkasova, 2009) 2*n* = 16, 28, 56*
*H. gmelinii* ACHK13082016	2*n* = 32; 0–3 B	
*H. gmelinii* NIK01082020	2*n* = 32	
*H. gmelinii* 54-19	2*n* = 16; 2*n* = 48; 0–2 B	
*H. setigerum* IOO23072005	2*n* = 32; 0–1 B	2*n* = 14* 2*n* = 32 (Plennik and Rostovtseva, 1977 2*n* = 48 (Gatsuk, 1967)
*H. setigerum* AKZ18072016	2*n* = 32; 0–2 B	
*H. grandiflorum* 32-10	2*n* = 16	2*n* = 16 (Philippov et al., 2008; Cherkasova, 2009)
*H. grandiflorum* 98-11	2*n* = 16	
*H. razoumovianum* SBR20072006	2*n* = 16	2*n* = 16 (Philippov et al., 2008; Cherkasova, 2009)
*H. zundukii* IOZ28072005	2*n* = 16	2*n* = 16 (Cherkasova, 2009)
*H. zundukii* IOO13072007	2*n* = 16; 0–5 B	

*Tropicos.org (Tropicos, 2022). Missouri Botanical Garden. IPCN Chromosome Reports.

**FIGURE 4:**
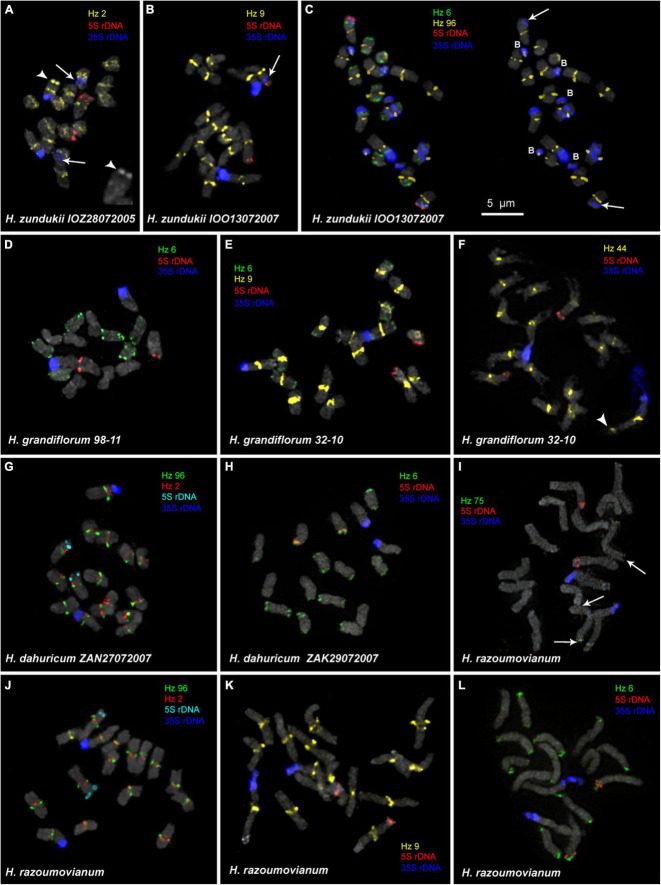
FISH-based localization of 5S rDNA, 35S rDNA, and satellite repeats on the metaphase spreads of the studied accessions of *H. zundukii* **(A–C)**, *H. grandiflorum* **(D–F)**, *H. dahuricum* **(G–H)**, and *H. razoumovianum* **(I–L)**. The correspondent probes and their pseudocolors are specified next to the metaphase spreads. Arrows point to polymorphic sites of 35S rDNA of *H. zundukii* **(A–C)**. Heads of arrows point to the Hz 2 site on the satellite chromosome of *H. zundukii* **(A)** and the Hz 44 site on the satellite chromosome of *H. grandiflorum* **(F)**. The increased fragment of one homolog of SAT chromosome of *H. zundukii* **(A)** with a large terminal DAPI-band is presented right below (head of arrow). Arrows point to the Hz 75 sites on chromosomes of *H. razoumovianum*. B, B chromosomes. Bar—5 μm.

**FIGURE 5:**
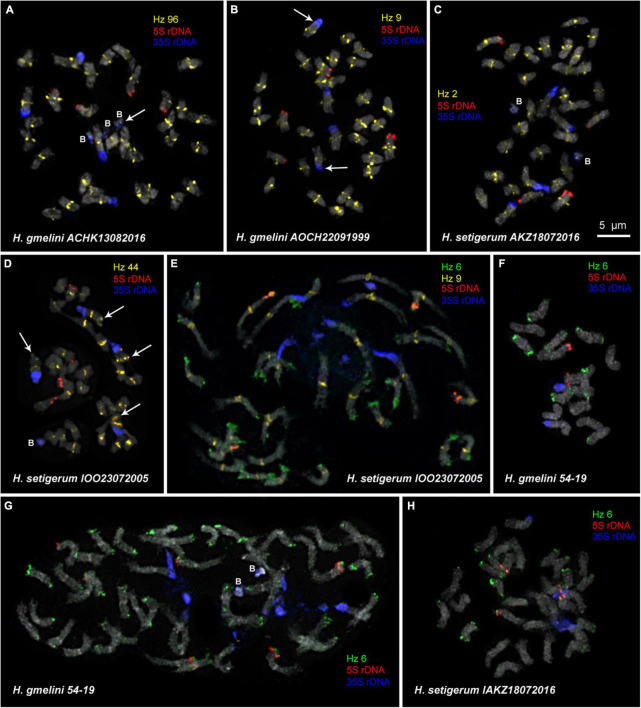
FISH-based localization of 5S rDNA, 35S rDNA, and satellite repeats in metaphase spreads of the studied accessions of *H. gmelinii* **(A,B,F,G)** and *H. setigerum* **(C–E,H)**. The correspondent probes and their pseudocolors are specified next to the metaphase spreads. Arrows point to the Hz 96 site on the B chromosome of *H. gmelinii* **(A)** and to small 5S rDNA sites (co-localized with 35S rDNA sites) on the satellite chromosomes of *H. gmelinii* **(B)**. Arrows point to the Hz 44 sites on the long arms of satellite chromosomes of *H. setigerum* **(D)**. B, B chromosomes. Bar—5 μm.

**FIGURE 6:**
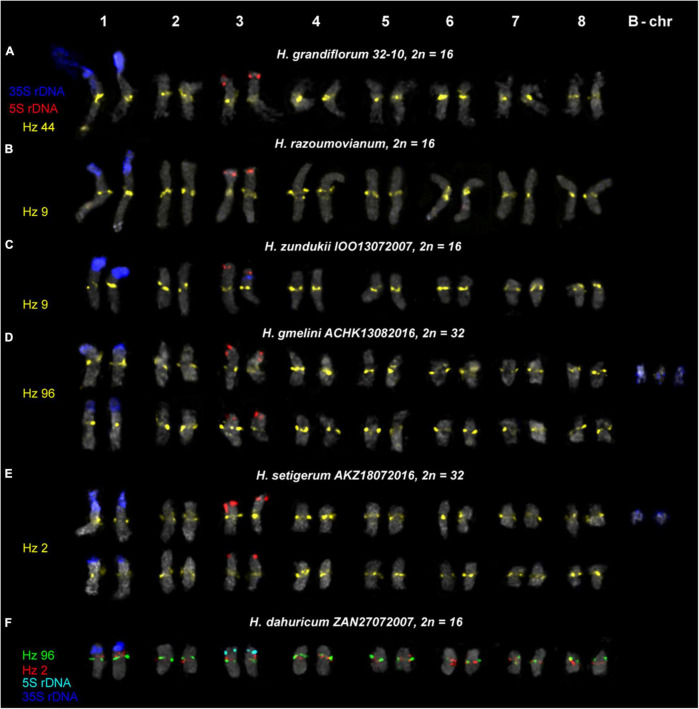
Karyotypes of the studied accessions of *H. grandiflorum* **(A)**, *H. razoumovianum* **(B)**, *H. zundukii* **(C)**, *H. gmelinii* **(D)**, *H. setigerum* **(E)**, and *H. dahuricum* **(F)** after FISH with 5S rDNA, 35S rDNA and satellite repeats (the same metaphase plates as in Figures 3, 4). The correspondent probes and their pseudocolors are specified on the left.

**FIGURE 7:**
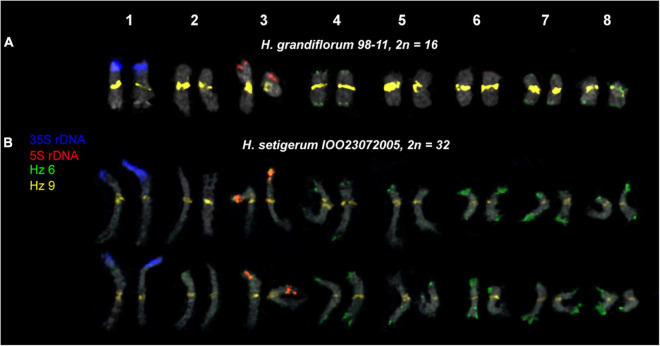
Karyotypes of the studied accessions of *H. grandiflorum* **(A)** and *H. setigerum* **(B)** after FISH with 5S rDNA, 35S rDNA, and satellite repeats Hz 6 and Hz 9 (the same metaphase plates as in Figures 3, 4). The correspondent probes and their pseudocolors are specified on the left.

**FIGURE 8:**
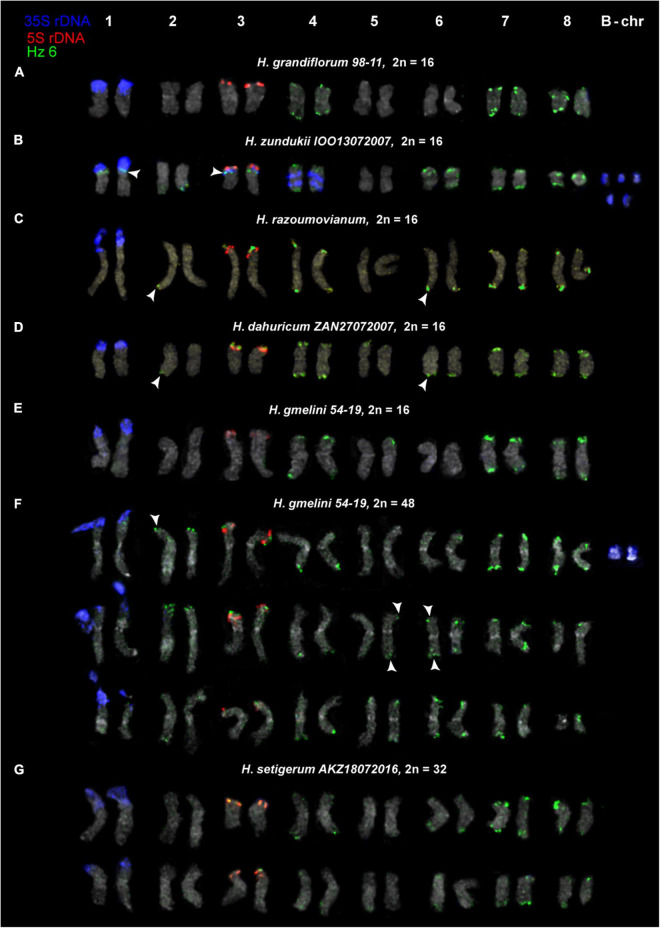
Karyotypes of the studied accessions of *H. grandiflorum* **(A)**, *H. zundukii* **(B)**, *H. razoumovianum* **(C)**, *H. dahuricum* **(D)**, *H. gmelinii* **(E,F)**, and *H. setigerum* **(G)** after FISH with 5S rDNA, 35S rDNA and satellite repeat Hz 6 (the same metaphase plates as in Figures 3, 4). Heads of arrows point to polymorphic sites of Hz 6. The correspondent probes and their pseudocolors are specified on the left.

We examined the chromosomal organization in six species of the sect. *Multicaulia* using the karyotype analysis, including the location of tandem repeated sequences (Figures 4, 5). For FISH mapping, we use 35S rDNA, 5S rDNA, and also oligonucleotide probes (Hz 2, Hz 6, Hz 9, Hz 44, Hz 59, Hz 75, and Hz 96) designed based on seven DNA repeats identified in *H. zundukii*, which exhibited high sequence homology with five DNA repeats of *H. grandiflorum* and six DNA repeats of *H. dahuricum* (Table 2 and Supplementary Table 1). Based on the morphology of chromosomes, and also chromosomal distribution of 35S rDNA, 5S rDNA, and the tandem DNA repeats, karyograms of these species were constructed (Figures 6, 7, 8).

We found that the karyotypes of the diploid (2*n* = 16) species *H. grandiflorum*, *H. zundukii*, *H. dahuricum*, and *H. razoumovianum* were mostly similar in chromosome morphology and also localization of clusters of 35S rDNA (chromosome pair 1) and 5S rDNA (chromosome pair 3) (Figures 4, 6). In *H. zundukii* accession of IOO13072007, a different number of additional minor 35S rDNA sites (polymorphic sites) was revealed in distal regions of chromosome pair 3 adjacent to 5S rDNA clusters (Figures 4B,C, 8B). In some karyotypes, the minor 35S rDNA site was observed only on one homolog of chromosome pair 3 (Figures 4B, 6C). A double hybridization site of 35S rDNA was detected in the pericentromeric region and also in the median part of the long arms of chromosome pair 4 (Figures 4C, 8B) (in *H. zundukii* IOO13072007) or only in the median region of the long arms of chromosome pair 4 (in *H. zundukii* IOZ28072005) (Figure 4A).

Karyotypes of closely related *H. gmelinii* and *H. setigerum* were represented by two similar sets of chromosomes with the same patterns of 35S and 5S rDNA localization indicating their tetraploid origin (2*n* = 4x = 32) (Figures 5A–E,H). In one *H. gmelinii* specimen from the Altai Mountains and also in all examined *H. setigerum* specimens, minor polymorphic 5S rDNA sites co-localized with 35S rDNA sites were revealed on one SAT chromosome pair 1 (Figure 5B). In one *H. gmelinii* specimen grown on a trial plot in Yakutsk (Republic of Sakha), diploid and hexaploid cytotypes were revealed (Figures 5F,G), which was confirmed by high morphological similarity of patterns of chromosomal distribution of main clusters of 35S rDNA and 5S rDNA in diploid (2*n* = 2x = 16) chromosome set and in each chromosome sets of hexaploid (2*n* = 6x = 48) species.

In some karyotypes of polyploid species *H. gmelinii* and *H. setigerum*, and also in diploid *H. zundukii*, we observed small supernumerary chromosomes (B chromosomes). They were about 1 μm in length, which demonstrated uncertain morphology and contained DAPI-positive regions. The number of such chromosomes in metaphase cells of the individual plants as well as in the populations could vary from 0 to 3 (in *H. gmelinii* and *H. setigerum*) or from 0 to 5 (in *H. zundukii*). We detected dispersed 35S rDNA sites along the entire length of these supernumerary chromosomes. Also, polymorphic sites of Hz 96 were revealed on some B chromosomes of *H. zundukii* (Figure 4C) and *H. gmelinii* (Figure 5A).

In all studied species of the sect. *Multicaulia*, we explored chromosomal localization of the selected satellite DNAs (refer to Supplementary Table 1). A total of four satDNA repeats (Hz 9, Hz 2, Hz 44, and Hz 96) demonstrated pericentromeric localization on chromosomes of all studied species (Figures 4, 5). Both Hz 96 (the longest pericentromeric tandem DNA) and Hz 9 (the most widely represented DNA repeat) exhibited large pericentromeric sites. In one specimen of *H. zundukii* (Figure 4A), the site of Hz 2 was detected in the region of the large terminal DAPI band of one homolog of SAT chromosome pair 1. FISH patterns of Hz 96 and Hz 2 repeats in the chromosomes of *H. dahuricum* and *H. razoumovianum* demonstrated that these repeats were localized separately (near each other) in the pericentromeric chromosome regions (Figures 4G,J, 6F). The localization of pericentromeric repeats in karyotypes of the studied diploid and polyploid species made it possible to clarify the morphology of chromosomes (Figures 6, 7). In *H. grandiflorum* and *H. setigerum*, minor polymorphic sites of Hz 44 were localized in the pericentromeric and terminal regions of the long arms of chromosome 1 (Figures 4F, 5D).

Hz 75 exhibited polymorphic sites in the terminal regions of several chromosomes in all studied species, e.g., in *H. razoumovianum* (Figure 4I and Supplementary Figure 1). Also, polymorphic sites of Hz 59 were detected in terminal regions of chromosomes of the most studied species except *H. grandiflorum* (Supplementary Figure 1).

For all studied species, the generalized idiograms showing chromosomal distribution of the Hz 6 markers, 35S rDNA, and 5S rDNA were constructed (Supplementary Figure 2). Polymorphic sites of Hz 6 were revealed by FISH in the subtelomeric regions of several chromosomes in all studied species, including *H. grandiflorum* (Figures 4, 5). In all diploid species, including *H. gmelinii* with 2*n* = 2x = 16, major Hz 6 sites were detected in the subtelomeric regions of both arms of chromosome pairs 4, 7, and 8 (Figures 7, 8). In karyotypes of *H. razoumovianum*, *H. dahuricum*, and *H. zundukii*, additional Hz 6 sites were detected in chromosome pairs 2 and 6, and also in the chromosome pair 3 near the 5S rDNA clusters (Figures 8B–D). In *H. zundukii*, polymorphic sites were also detected in the secondary constriction regions of chromosome pair 1 (Figure 6B). In *H. gmelinii* and *H. setigerum*, major sites of Hz 6 were localized in the subtelomeric regions of both arms of chromosome pairs 4, 7, and 8. In hexaploid *H. gmelinii* with 2*n* = 6x = 48, polymorphic sites of Hz 6 were detected on chromosome pairs 2, 3, 5, and 6 (Figure 8F).

## Discussion

The widespread genus ***Hedysarum*** involves many valuable medicinal and fodder species (Fedtschenko, 1948; Sa et al., 2010; Dong et al., 2013; Liu Y. et al., 2019). Most species of the sect. ***Multicaulia*** are distributed in southern Siberia and Central Asia. The range of the studied species of the sect. ***Multicaulia*** is divided into two non-overlapping areas: (1) ***H. grandiflorum***, ***H. razoumowianum*,** and ***H. gmelinii*** grow in the East-European region, and ***H. zundukii***, ***H. dahuricum***, ***H. setigerum*,** and ***H. gmelinii*** distributed in the South-Siberian region, i.e., ***H. gmelinii*** can grow in both regions and its range overlaps with all studied species. ***Hedysarum grandiflorum***, ***H. razoumowianum***, and ***H. zundukii*** are considered to be rare and endangered taxa (KMK Publ, 2008). As shown on the constructed map, the species of the sect. ***Multicalia*** can occupy both wide and very narrow areas. ***Hedysarum grandiflorum*** is a rare but wide-ranging species, whereas both ***H. razoumowianum*** and ***H. zundukii*** occupy narrow areas. The ranges of ***H. setigerum*** and ***H. gmelinii*** are partially overlapped, and morphological similarities between these closely related species made controversial to their taxonomy (Fedtschenko, 1948; Kurbatsky, 1994; Sa et al., 2010).

It was previously reported that *x* = 8 is the basic chromosome number for species of the sect. *Multicaulia* (Arslan et al., 2012; Choi and Ohashi, 2003; Duan et al., 2015; Liu et al., 2017; Nafisi et al., 2019). In this study, we confirmed this basic chromosome number for the studied species, including *H. dahuricum*, which karyotype was explored for the first time. In species from the sect. *Multicaulia*, variations in the number of chromosomes in karyotypes were previously described with the use of monochrome staining (Gatsuk, 1967; Plennik and Rostovtseva, 1977; Malahova and Kurbatsky, 1992; Philippov et al., 2008; Cherkasova, 2009). We also detected diploid, tetraploid, and hexaploid karyotypes in *H. gmelinii* specimens from different geographical regions. At the same time, in the studied *H. setigerum* specimens, we revealed only tetraploid karyotypes although variations in the number of chromosomes in karyotypes (14, 32, and 48) were previously reported for this species (Gatsuk, 1967; Plennik and Rostovtseva, 1977). Moreover, we revealed small supernumerary B chromosomes in *H. zundukii, H. gmelinii*, and *H. setigerum*. B chromosomes were previously revealed in the karyotype of a diploid Siberian species *H. sangilense* (Krogulevich and Rostovtseva, 1984). These chromosomes are characterized by a mosaic distribution within the population as well as in individual plants (Camacho et al., 2000; Houben et al., 2014; D’Ambrosio et al., 2017). In our study, the number of B chromosomes also varied in karyotypes of *H. zundukii*, *H. gmelinii*, and *H. setigerum*. Besides, we often detected dispersed 35S rDNA and, sometimes, revealed satDNA hybridization signals on these supernumerary chromosomes. It was earlier reported that B chromosomes could contain rDNA genes and/or tandem repeats (D’Ambrosio et al., 2017; Marques et al., 2018; Ebrahimzadegan et al., 2019). The performed comparative molecular cytogenetic analysis and also detection of B chromosomes in the karyotypes of *H. zundukii*, *H. gmelinii*, and *H. setigerum* allowed us to establish the number of chromosomes in the main sets of these species.

In karyotypes of all studied diploid species, we revealed one chromosome pair bearing a major 35S rDNA cluster and one pair with a 5S rDNA cluster. At the same time, the chromosomal distribution of these molecular markers differed from that revealed in the species of the sect. *Hedysarum* (Yurkevich et al., 2021). The analysis of chromosome morphology, as well as patterns of chromosomal distribution of 35S rDNA, allowed us to confirm the tetraploid and/or hexaploid nature of *H. setigerum* and *H. gmelinii* specimens and also demonstrate close genome relationships among studied species of the sect. *Multicaulia.*

The performed comparative bioinformatic analysis of repeatomes of the species from the sect. *Multicaulia* in this study demonstrated a high similarity in repeatome composition of *H. grandiflorum*, *H. zundukii*, and *H. dahuricum* and also established common features in their repeatomes. In genomes of eukaryotes, retrotransposons (Class I) are the most abundant transposable elements. Within the legume family, their composition can vary in different species due to the predominant number of Ty1 Copia (Istvánek et al., 2014; Jegadeesan et al., 2021) or Ty3-Gypsy elements (Kreplak et al., 2019; Lonardi et al., 2019). In the studied species of the sect. *Multicaulia*, mobile elements of Class I made up the majority of their repetitive DNA (20–24%), and Ty3-Gypsy retroelements were almost 1.5–2 times more abundant when compared to Ty1-Copia elements. Retrotransposons are known to be replicated with the copy and paste mechanisms, and they can be accumulated in nuclear genomes (Bennetzen and Wang, 2014). The plant species having small genome sizes contain fewer LTR retrotransposons compared to plants with large genomes (Vitte and Bennetzen, 2006; Bennetzen and Wang, 2014; Wang et al., 2021). Therefore, variations in genome sizes, observed within legume family, can be explained by variability in the content of LTR retrotransposons (Macas et al., 2015; Ellis and Vershinin, 2020). In diploid *Hedysarum* species with 2*n* = 2x = 16, the amount of nuclear DNA ranged within 2C = 1.26–3.4 pg (Benhizia et al., 2021), which was relatively small for plants (Vitte and Bennetzen, 2006; Bennetzen and Wang, 2014; Wang et al., 2021). This fact was quite consistent with the ratio of low-copy and repetitive DNA sequences detected in genomes of the studied species of the sect. *Multicaulia.*

In different satellite DNA families, a rather high rate of genomic changes was revealed, and satellite DNAs can be either species-specific or common to a certain group of related species (Garrido-Ramos, 2015). Most Fabaceae species are characterized by a large number of various satellite repeats (Neumann et al., 2012; Macas et al., 2015; Ávila Robledillo et al., 2020). Despite the fact that the number of identified tandem DNAs differed in *H. grandiflorum*, *H. zundukii*, and *H. dahuricum*, the main set of common tandem DNA repeats was homologous and their monomer sequences were mostly identical in length.

Thus, the repeatomes of the studied related species of the sect. *Multicaulia* have more common satellite repeats than species-specific ones, which could be related to their common origin. The revealed close relationship between genomes of the species from two subsections *Multicaulia* (*H. dahuricum*, *H. razoumovianum*, *H. setigerum*, and *H. gmelinii*) and *Subacaulia* (*H. grandiflorum* and *H. zundukii*) confirms the results of phylogenetic studies reported earlier (Choi and Ohashi, 2003; Duan et al., 2015; Liu Y. et al., 2019).

SatDNA is often associated with heterochromatin and localized in the certain chromosome regions, which allows it to be studied using various cytogenetic techniques, such as FISH (Mehrotra and Goyal, 2014; Biscotti et al., 2015). In some Fabaceae species, several (2–12) satellite DNA repeats were previously localized in the pericentromeric regions of chromosomes, and most of these repeats were species-specific (Ávila Robledillo et al., 2020). According to our FISH results, four common tandem DNA repeats (Hz 9, Hz 2, Hz 44, and Hz 96) presented similar pericentromeric co-localization on the chromosomes of the studied species, which allowed us to clarify the chromosome morphology and also confirm a close genomic relationship between these species.

Hz 75, Hz 59, and Hz 6 repeats were FISH mapped predominantly in the terminal regions of chromosomes in the studied species except *H. grandiflorum* in the genome of which Hz 59 was not detected by TAREN. At the same time, Hz 75 and Hz 6, which were FISH mapped on chromosomes of *H. dahuricum* and *H. grandiflorum*, were also not detected by TAREN in their repeatomes. This might be due to some features of the used sequencing method, subsequent bioinformatic processing, as well as to the prevalence of satDNA in the genomes of these species. These results show that cytogenetic studies are important for the investigation of plant genomes, since they refine the information obtained after bioinformatics analysis.

SatDNA is considered to be involved in the main processes of formation of the most important chromosomal structures, e.g., DNA packaging and chromatin condensation, and it was reported to represent recombination “hotspots” of genome reorganization (Plohl et al., 2012; Biscotti et al., 2015). The content of satDNA can vary in plant genomes even between generations, which results in high polymorphism in the length of satellite arrays (Plohl et al., 2012; Macas et al., 2015; Ávila Robledillo et al., 2020). Currently, the patterns of distribution of satDNAs as chromosome markers are widely used to detect rearrangements, to identify chromosomes and subgenomes in karyotypes of diploid and polyploid plants, as well as to study the paths of chromosomal evolution of related taxa (Samoluk et al., 2017; Belyayev et al., 2019; Liu Q. et al., 2019; Mata-Sucre et al., 2020). In the studied species from the section *Multicaulia* of the genus *Hedysarum*, FISH patterns of chromosomal distribution of Hz 6, both separately or together with any of the pericentromeric probes (Hz 9, Hz 2, Hz 96, and Hz 44), were chromosome-specific, which allowed us to identify all chromosome pairs in karyotypes. At the same time, these molecular chromosomal markers did not reveal chromosomal rearrangements in the karyotypes of the studied species, as it was earlier found in other species (Belyayev et al., 2019; Amosova et al., 2021; Waminal et al., 2021). On chromosomes of the studied species, Hz 6 presented a specific distribution pattern with permanent sites, which were localized in the subtelomeric regions of three pairs of chromosomes, and also, several polymorphic sites were detected on the remaining chromosome pairs. Thus, the permanent sites of Hz 6 in combination with any of the pericentromeric sites of Hz 9, Hz 2, Hz 96, or Hz 44 were the effective molecular markers for chromosome identification. Polymorphic sites of Hz 6, Hz 75, and Hz 59, detected in the terminal regions of several chromosomes, were valuable molecular chromosome markers to analyze intra- and interspecies chromosomal variabilities in the studied species. Moreover, the tandem DNAs Hz 9, Hz 2, Hz 96, Hz 44, and Hz 6 could be useful for comparative cytogenetic studies to clarify the evolutionary relationships within *Hedysarum.*

The species *H. setigerum* and *H. gmelinii* are rather similar in morphological characters, and taxonomists identify *H. setigerum* either as a separate species (Fedtschenko, 1948) or as a subspecies of *H. gmelinii* (Kurbatsky, 1994; Sa et al., 2010). In Southern Siberia, both species have overlapping ranges and occupy similar habitats (Fedtschenko, 1948; Malyshev et al., 2012). In both *H. gmelinii* and *H. setigerum*, the performed FISH analysis demonstrated pattern similarity in chromosomal distribution of major 35S rDNA and 5S rDNA clusters, minor 5S rDNA sites, and also tandem DNA repeats. Our findings are consistent with the results of the ISSR analysis, which indicated a close relationship between *H. setigerum* and *H. gmelinii* (Zvyagina et al., 2016), and also with the reported earlier suggestion that *H. setigerum* was most likely a subspecies of *H. gmelinii* (Kurbatsky, 1994; Sa et al., 2010; Zvyagina et al., 2016). *H. dahuricum* has overlapping areas with *H. gmelinii* and *H. setigerum*, and it was included in the *Hedysarum gmelinii* group (Sa et al., 2010). In our study, FISH-based patterns of chromosomal localization of Hz 6 in *H. dahuricum* were similar to those revealed in *H. setigerum* and *H. gmelinii*, which was consistent with previously reported data on a close relationship between *H. dahuricum* and the yellow-flowered form of *H. gmelinii* (Fedtschenko, 1948). Morphologically well-differentiated, *H. razoumovianum* and *H. grandiflorum* occupy the overlapped region within Eastern Europe though they are considered to belong either to different sections (Fedtschenko, 1948) or to subsections *Multicaulia* and *Subacaulia* (Choi and Ohashi, 2003). Our findings demonstrate that the karyotypes of Eastern European *H. razoumovianum* are rather similar to karyotypes of South Siberian species belonged to the subsect. *Multicaulia*. Previously, the morphological similarity between *H. gmelinii* and *H. razoumovianum* was described, which was believed to indicate their relationship and the common geographical origin from the Asian ancestor closely related to *H. gmelinii* (Gorchakovsky, 1969).

It was suggested that morphological similarity of the Eastern European species *H. grandiflorum* (subsect. *Subacaulia*) and the South Siberian *H. zundukii* (subsect. *Subacaulia*) could be related to their growth in similar conditions on carbonate rocks (Peshkova, 2001). The relict species *H. zundukii* occupies a very narrow range, and it was reported that its discrete distribution within this range was due to its adaptation to certain environmental conditions (Karnaukhova et al., 2008). Nevertheless, for the natural populations of *H. zundukii*, some heterogeneities of morphological characters were described (Cherkasova, 2008). On the other hand, *H. zundukii* exhibits low polymorphism in seed storage proteins, which is typical for species with a small range and small population size (Konichenko and Selytina, 2011).

All South Siberian species of the subsect. *Subacaulia*, including *H. zundukii*, are believed to be related to *H. gmelinii* (Kurbatsky, 1994). Among the studied diploid (2*n* = 16) species, *H. zundukii* exhibited the greatest variety of tandem DNA repeats and the most significant intraspecific variability in patterns of chromosomal localization of molecular markers. Moreover, the minor 35S rDNA sites as well as maximum sites of Hz 6 were detected on chromosomes of *H. zundukii*. At the same time, according to the patterns of chromosomal localization of Hz 6 repeat, *H. zundukii* (subsect. *Subacaulia*) was more similar to *H. setigerum* and *H. gmelinii* (subsect. *Multicaulia*) compared to *H. grandiflorum* (subsect. *Subacaulia*).

Considering that *H. zundukii*, *H. setigerum*, and *H. gmelinii* occupy the same areas of the western coast of Lake Baikal, and the plants with intermediate (between *H. zundukii* and *H. gmelinii*) traits have already been found (Kurbatsky and Malahova, 1992), it is likely that spontaneous interspecific hybridization could increase the genetic intraspecific diversity in the relict species *H. zundukii*.

Thus, our comprehensive comparative study of genomes of six species of the sect. *Multicaulia* of the genus *Hedysarum* detected a close relationship among their genomes (regardless of the region of their growth and the range size), indicating a common origin of these species. At the same time, based on the analysis of intra- and interspecific variabilities in patterns of chromosomal distribution of molecular markers (35S rDNA, 5S rDNA, and tandem DNA repeats), we could subdivide the studied species into four groups: (1) *H. zundukii* (subsect. *Subacaulia*), (2) *H. setigerum* and *H. gmelinii* (subsect. *Multicaulia*), (3) *H. dahuricum*, *H. razoumovianum* (subsect. *Multicaulia*), and (4) *H. grandiflorum* (subsect. S*ubacaulia*). Also, a comparative analysis of genomes of the studied species allowed us to detect tetraploid and hexaploid forms in *H. setigerum* and *H. gmelinii* and also confirm the taxonomic status of *H. setigerum* as a subspecies of *H. gmelinii*. Our findings indicate the validity of combining the sections *Multicaulia* and *Subacaulia* into one section *Multicaulia*.

## Conclusion

The comparison of repeatomes of *H. grandiflorum*, *H. dahuricum*, and *H. zundukii* revealed species-specific differences in genome composition, and also high sequence similarity in the identified satDNAs. FISH mapping of the identified tandem DNA repeats on chromosomes of six *Hedysarum* species allowed us to assess genome diversity within the section *Multicaulia* and determine new effective molecular chromosome markers especially important for comparative karyotypic studies. In all studied species, we revealed intra- and interspecific variabilities in patterns of chromosomal distribution of the detected chromosome markers and constructed species karyograms. In *H. gmelinii* and *H. setigerum*, similar subgenomes were detected confirming the polyploid status of their genomes. Our findings demonstrated a close relationship among genomes of six studied species indicating their common origin and also confirmed the taxonomic status of *H. setigerum* as a subspecies of *H. gmelinii* as well as the validity of combining the sections *Multicaulia* and *Subacaulia* into one section *Multicaulia*.

## Data Availability Statement

The datasets presented in this study can be found in online repositories. The name of the repository and accession number can be found below: National Center for Biotechnology Information (NCBI) BioProject database under accession number PRJNA811959 (https://www.ncbi.nlm.nih.gov/bioproject/PRJNA811959).

## Author Contributions

OY and OM contributed to methodology and conceptualization. TS, SZ, IS, SS, and NS contributed to formal analysis. IS and NS contributed to plant materials. OY, TS, SZ, IS, NS, SS, AA, and OM contributed to investigation and contributed to writing—original draft. OM contributed to supervision. OY, TS, SZ, IS, NS, SS, and AA contributed to visualization. OY, AA, and OM contributed to writing, reviewing, and editing the manuscript. All authors contributed to the article and approved the submitted version.

## Conflict of Interest

The authors declare that the research was conducted in the absence of any commercial or financial relationships that could be construed as a potential conflict of interest.

## Publisher’s Note

All claims expressed in this article are solely those of the authors and do not necessarily represent those of their affiliated organizations, or those of the publisher, the editors and the reviewers. Any product that may be evaluated in this article, or claim that may be made by its manufacturer, is not guaranteed or endorsed by the publisher.

## Footnotes

1
http://www.ncbi.nlm.nih.gov/bioproject/811959

2
https://repeatexplorer-elixir.cerit-sc.cz/galaxy/
