# Supplementary material for: Integration of Genomic and Cytogenetic Data on Tandem DNAs for Analyzing the Genome Diversity Within the Genus Hedysarum L. (Fabaceae)
Source: Front Plant Sci. 2022 Apr 29;13:865958. doi: 10.3389/fpls.2022.865958 (PMC9101955; doi:10.3389/fpls.2022.865958)
Supplement: Supplementary file 1 [file Data_Sheet_1.PDF]

Supplementary Material

1 Supplementary Data

Supplementary Table 1. List of the oligonucleotide FISH probes.

Tandem Repeat	Oligo FISH probe name/ length, bp	Oligo FISH probe sequence
**Hz 6**	Hz 6_1/20	AGACTCAAGGGATGGCAATG
	Hz 6_2/20	GCCCAACTCTTGCAAAGAAG
	Hz 6_3/20	AAGGCCTACAATGCATGGAG
**Hz 9**	Hz9_1/90	GTCATTCATGGCCTTAATAGTGGAGGAATAGAAAGGTTTCTAGTT GGACTCTAGACTAGCCAAATTTCATAGAATTCCTTACCTTAGCCA
	Hz9_2/90	TTAAGGTATATACTTCTCTTAGTAATTCTTGCCCAAAACGAAGTC TATAGTACTCACACTAGGTATTTTTATCTAAATACATGGTTTGGT
**Hz 2**	Hz 2/50	GTCCAGGTTAGTGGTCATCTAGTGTTAGGAACCTACAGTTGACCT AATTG
**Hz 44**	Hz 44_1/20	GCCAAAATAGCCGAAACTCA
	Hz 44_2/20	CGGTTGACCGAGTCAAGAAT
**Hz 96**	Hz 96_1/30	TTATCTATAACAATATGTAAAGGGGATTCC
	Hz 96_2/20	TTGGAATGAACAATGGCAAA
**Hz 75**	Hz 75_1/20	ACCGTGGAAGAGACACGATC
	Hz 75_2/20	CCCCAACAGACGTTCTTTGT
**Hz 59**	Hz 59_1/20	ATATGACCGAACGCAGCTCT
	Hz 59_2/20	AGGAGTGAAGTGCTCCCAAA

Supplementary Table 2. Proportion of Major Repetitive DNA Sequences Identified in Genomes of the Studied *Hedysarum* Species.

Repeat Name	Genome proportion (%)		
	H. grandiflorum	*H. dahuricum*	*H. zundukii*
**Retrotransposons (Class I)**	**24.23**	**22.47**	**19.95**
**Ty1 Copia**	**9.1**	**7.1**	**6.78**
Ale	0.21	0.15	0.16
Angela	0.67	0.77	0.69
Bianca	0.59	0.25	0.18
SIRE	6.55	5.3	5.27
TAR	0.68	0.46	0.42
Tork	0.4	0.17	0.06
**Ty3-Gypsy**	**14.87**	**14.66**	**13**
non-chromovirus Athila	4.12	3.63	3.54
non-chromovirus Tat- Ogre	0.61	0.83	0.71
non-chromovirus Tat-Retand	1.35	1.1	1.01
chromovirus CRM	0.13	0.07	0.1
chromovirus Tekay	8.66	9.03	7.64
**LINE**	**0.23**	**0.18**	**0.17**
**Pararetrovirus**	**0.03**	**0.02**	**-**
**Unclassified LTR elements**	**-**	**0,51**	**-**
**Transposons (Class II)**	**2.04**	**2.55**	**2.28**
Cacta	1.85	2.28	2.07
MuDR_Mutator	0.19	0.27	0.21
**rDNA**	1.08	1.45	2.6
**Unclassified repeat**	8.27	8.55	9.95
**DNA satellite**	5.09	2.68	4.08
**Organelle**	7.83	9.18	4.58
**Repetitive DNA**	**48.54**	**46.88**	**43.44**
**Putative satellites**	5 high confident<br>5 low confident	5 high confident<br>7 low confident	5 high confident<br>10 low confident

2. Supplementary Figures

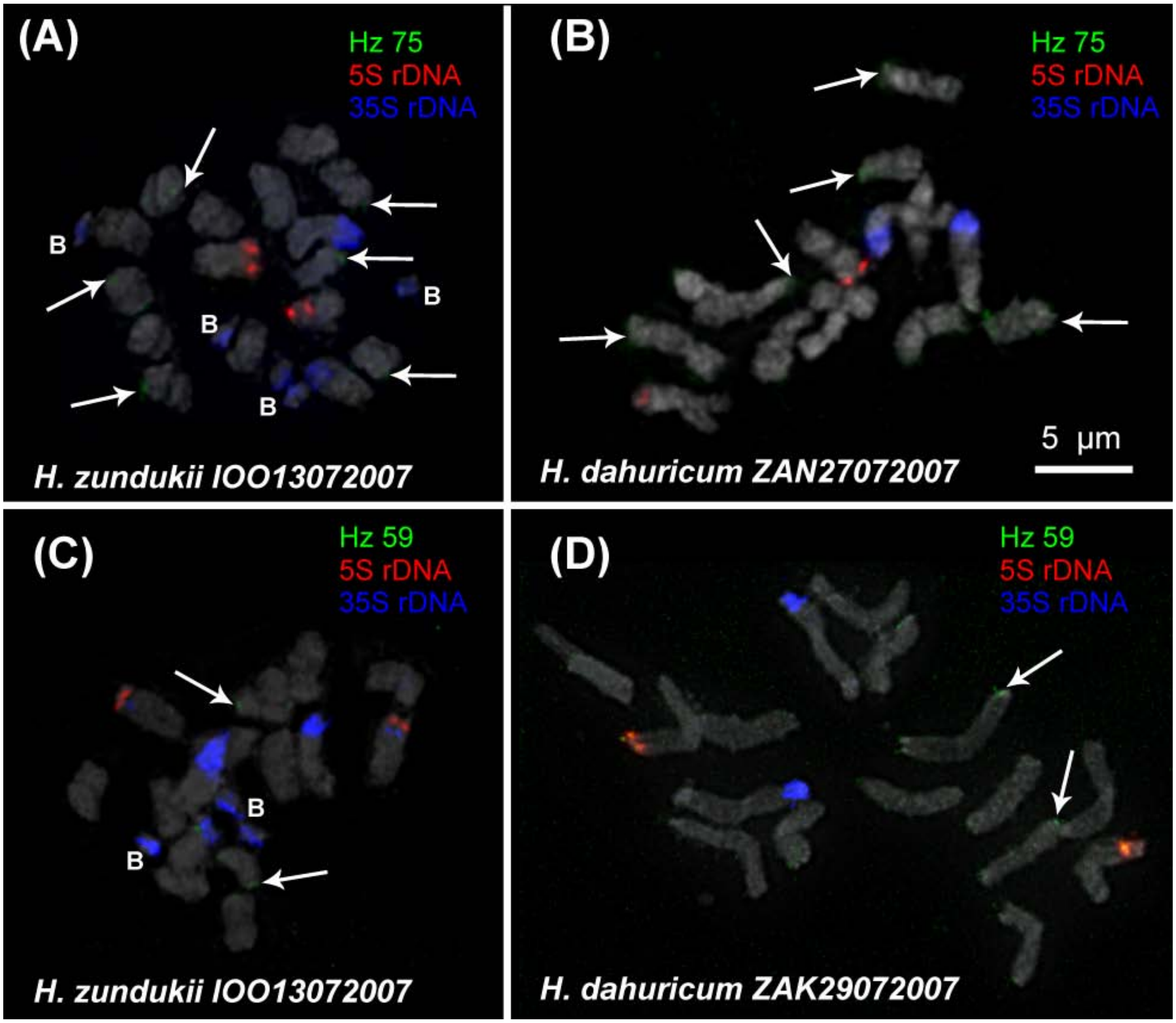

Supplementary Figure 1. FISH-based localization of 5S rDNA, 35S rDNA and satellite repeats in metaphase spreads of the studied accessions of *H. zundukii* (A, C) and *H. dahuricum* (B, D). The correspondent probes and their pseudocolors are specified next to the metaphase spreads. Arrows point to sites of Hz 75 and Hz 59.

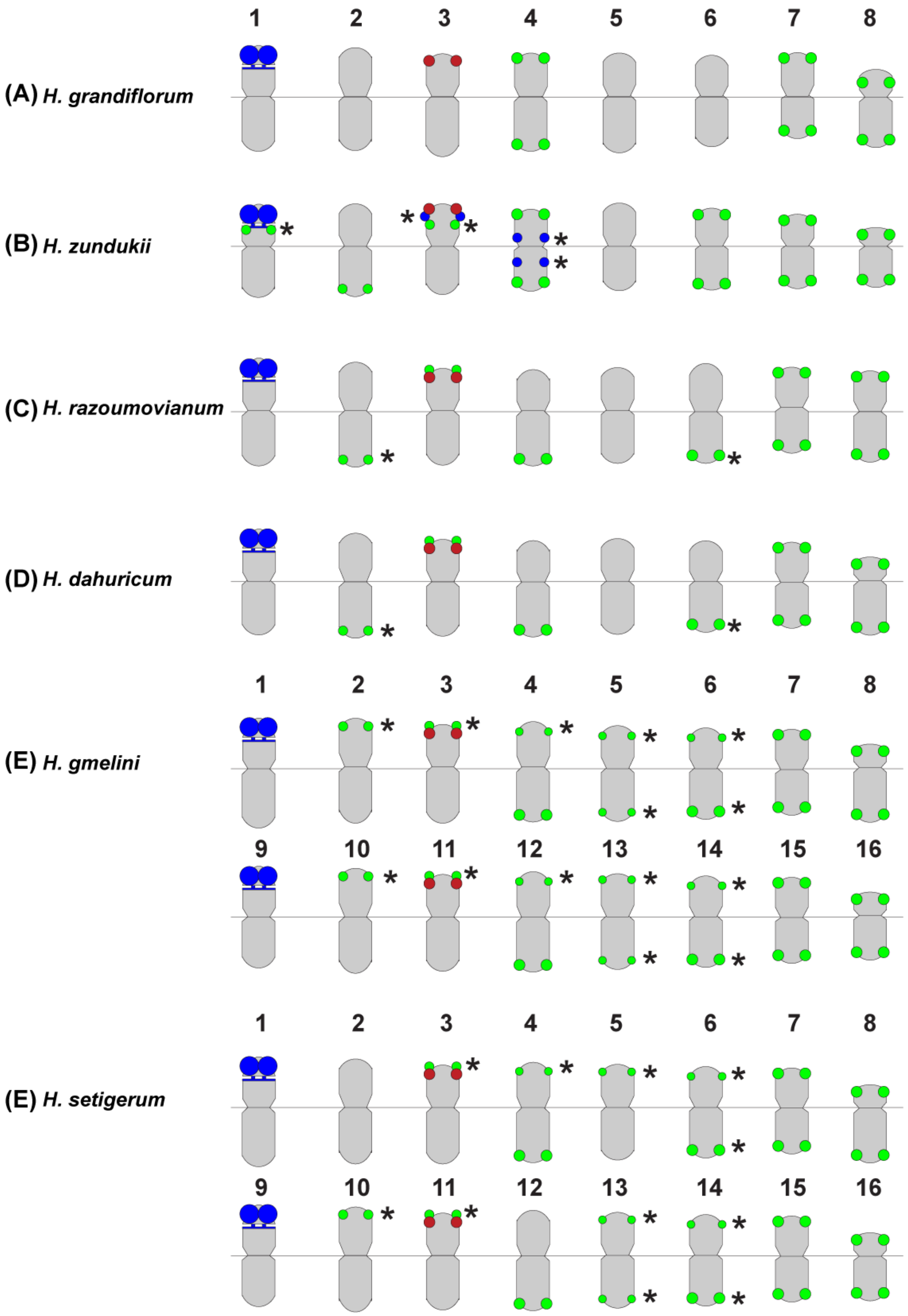

Supplementary Figure 2. Generalized idiograms of *Hedysarum* chromosomes showing the chromosomal distribution of the examined markers: Hz 6 (green), 35S rDNA (blue), and 5S rDNA (red). Asterisks indicate polymorphic sites.
